# Altered intragenic DNA methylation of *HOOK2* gene in adipose tissue from individuals with obesity and type 2 diabetes

**DOI:** 10.1371/journal.pone.0189153

**Published:** 2017-12-11

**Authors:** Sandra Rodríguez-Rodero, Edelmiro Menéndez-Torre, Gustavo Fernández-Bayón, Paula Morales-Sánchez, Lourdes Sanz, Estrella Turienzo, Juan José González, Ceferino Martinez-Faedo, Lorena Suarez-Gutiérrez, Jessica Ares, Lucia Díaz-Naya, Alicia Martin-Nieto, Juan L. Fernández-Morera, Mario F. Fraga, Elías Delgado-Álvarez

**Affiliations:** 1 Endocrinology and Nutrition Department, Hospital Universitario Central de Asturias (HUCA), Asturias, Spain; 2 Cancer Epigenetics Laboratory, Institute of Oncology of Asturias (IUOPA), HUCA, Universidad de Oviedo, Asturias, Spain; 3 Endocrinology, Nutrition, Diabetes and Obesity Unit, Instituto de Investigación Sanitaria del Principado de Asturias (ISPA), Oviedo, Asturias, Spain; 4 Surgery Department, Hospital Universitario Central de Asturias, Asturias, Spain; 5 Centro de Investigación en Nanomateriales y Nanotecnología (CINN), El Entrego, Asturias, Spain; 6 Medicine Department, Universidad de Oviedo, Asturias, Spain; Institut de genomique, FRANCE

## Abstract

**Aims/Hypothesis:**

Failure in glucose response to insulin is a common pathology associated with obesity. In this study, we analyzed the genome wide DNA methylation profile of visceral adipose tissue (VAT) samples in a population of individuals with obesity and assessed whether differential methylation profiles are associated with the presence of type 2 diabetes (T2D).

**Methods:**

More than 485,000 CpG genome sites from VAT samples from women with obesity undergoing gastric bypass (n = 18), and classified as suffering from type 2 diabetes (T2D) or not (no type 2 diabetes, NT2D), were analyzed using DNA methylation arrays.

**Results:**

We found significant differential methylation between T2D and NT2D samples in 24 CpGs that map with sixteen genes, one of which, *HOOK2*, demonstrated a significant correlation between differentially hypermethylated regions on the gene body and the presence of type 2 diabetes. This was validated by pyrosequencing in a population of 91 samples from both males and females with obesity. Furthermore, when these results were analyzed by gender, female T2D samples were found hypermethylated at the cg04657146-region and the cg 11738485-region of HOOK2 gene, whilst, interestingly, male samples were found hypomethylated in this latter region.

**Conclusion:**

The differential methylation profile of the *HOOK2* gene in individuals with T2D and obesity might be related to the attendant T2D, but further studies are required to identify the potential role of *HOOK2* gene in T2D disease. The finding of gender differences in T2D methylation of *HOOK2* also warrants further investigation.

## Introduction

Obesity is frequently associated with the resistance of peripheral tissues (muscle and adipose) to the action of insulin. Because of a state of inflammation in adipose tissue infiltrated by inflammatory cells, secretions from these cells and fat derived adipokines promote the development of type 2 diabetes (T2D) [1–3]. Genome wide studies have led to the characterization of gene variants that are associated with an increased risk of T2D development, but their effect size is reduced when compared with traditional risk factors such as obesity, unhealthy diet or family history of diabetes [4].

Previous works have demonstrated alterations in methylation profiles of genes involved in the pathogenesis of obesity and T2D development [5]. For example, in subcutaneous adipose tissue from a group of 31 healthy men following exposure to exercise training [6], changes in methylation were found related to variations in mRNA expression in some of the T2D associated genes––*HHEX* (Hematopoietically-Expressed Homeobox Protein), *IGF2BP2* (Insulin-like Growth Factor 2 Binding Protein 2), *JAZF1* (JAZF Zinc Finger 1) and *TCF7L2* (Transcription Factor 7-like 2). Ribel-Madsen et al conducted genome-wide methylation studies in insulin responsive tissues (skeletal muscle and subcutaneous adipose tissue) in elderly monozygotic (MZ) twin pairs discordant for T2D [7]. Methylation differences were found on the promoter region of *CDKN2A* (Cyclin-Dependent Kinase Inhibitor 2A) and *HNF4A* (Hepatocyte Nuclear Factor 4 Alpha) genes, although greater differences were found in genome wide repetitive DNA sequences such as LINE-1. These results were complemented by Nilsson et al, who described 1410 differentially methylated CpG sites between MZ T2D discordant twins, including *KCNQ1* (Potassium Channel Voltage Gated KQT-like Subfamily Q, Member 1), *NOTCH2* (Neurogenic Locus Notch Homolog Protein 2), *TCF7L2* (Transcription Factor 7-like 2) and *THADA* (Thyroid Adenoma Associated) T2D related genes [8]. Recently, work by Chen et al has demonstrated that promoter hypermethylation of *NR4A1* (Nuclear Receptor Subfamily 4 Group A Member 1) was elevated in T2D human samples as well as in T2D murine models and that this hypermethylation was associated with a decrease in mRNA levels. They also found that lack of NR4A1 (Nuclear Receptor Subfamily 4 Group A Member 1) protein is related to an increment in DNMT1 expression and the blocking of insulin signaling in patients with T2D [9]. These results were supported by the genome-wide DNA methylation analysis undertaken by Volkov et al in subcutaneous adipose tissue samples [10], whose work demonstrated an association between genetic and epigenetic mechanisms in terms of an observed relationship between single nucleotide polymorphisms and methylation at CpG sites of genes involved in metabolic patterns associated with diabetes development.

The majority of T2D cases occur in the context of a metabolic syndrome leading to a chronic excess of energetic substrates and ectopic fat storage, insulin resistance, elevated levels of inflammatory cytokines and, ultimately, a decrease in insulin secretion and the apoptosis of pancreatic β cells. The studies referred to earlier, have demonstrated the DNA methylation of T2D susceptible genes in different tissues (blood, skeletal muscle and fat) which are frequently associated with T2D development. Most of these works were carried out on subcutaneous fat, although VAT, along with skeletal muscle and liver, is in fact the most suitable candidate for determining the involvement of gene methylation in T2D development. Visceral fat not only acts as an energy storage tissue, but also as an endocrine organ, which releases hormones and adipokines, that contribute to expand macrophage population in VAT, which also liberate inflammatory cytokines. As the fat depot increases, levels of these molecules also rise and since they modulate the action of insulin in muscle and liver insulin insensitivity may result. VAT tissue has high levels of lipogenesis and lipolysis activity. This can result in hyperlipidemia and glucose intolerance [11]. Moreover, excess free fatty acids lead to ectopic lipid accumulation and lipotoxicity in muscle, where they inhibit glucose uptake, causing insulin resistance in these tissues [12, 13]

Prior research has described genome-wide DNA methylation patterns and site-specific differences in CpG methylation of candidate genes associated with T2D in VAT [14–18]. The aim of the present study is to contribute to the description of the VAT methylome in humans, and to explore the impact of epigenetics in diabetes development.

## Materials and methods

### Ethics statement

The study protocol (n° 68/10) was approved by the Ethical Committee at the Hospital Universitario Central de Asturias (Asturias, Spain) and all participants provided written and oral informed consent. The study was conducted in accordance with the principles of the Helsinki Declaration for human research.

### Human tissue samples

**Discovery cohort**: a cohort of 18 (8 T2D and 10 NT2D) visceral adipose tissue (VAT) samples was obtained from patients (all female) from the Surgery and Endocrinology Departments at the Hospital Universitario Central de Asturias who underwent gastric bypass. Criteria for bariatric bypass surgery were BMI ≥35 and at least one obesity-related comorbidity (diabetes (n = 8), cardiovascular disease, CVD, (n = 9), non-alcoholic fatty liver disease (n = 4) or hypertension (n = 12)). The anthropometric and clinical characteristics of the samples are presented in **Table 1**.

**Table 1:** The anthropometric and clinical characteristics of the discovery cohort and the validation cohort.

	Discovery Cohort			Biological Validation Cohort		
Characteristics	NT2D	T2D	*p*-value	NT2D	T2D	*p*-value
**N(male/female)**	10 (0/10)	8(0/8)		55 (14/41)	36 (16/20)	
**Age (years)**	48.2 ± 9.16	50.3 ± 7.68	0.4562	41.5 ± 10.1	46.47 ± 11.54	**0.0358**
**BMI(kg/m**^**2**^**)**	50.48 ± 7.82	44.91 ± 3.8	0.0661	47.06 ± 8.92	49.06 ± 5.84	0.4482
**Fasting Plasma Glucose (mg/dl)**	118.20 ± 37.95	213 ± 85.44	**0.0144**	118.17 ± 38.43	166.5 ± 66.03	**0.0002**
**Total-Cholesterol (mg/dl)**	171.8 ± 30.8	216.33 ± 33.15	0.1682	192.73 ± 31.89	181.5 ± 39.65	0.3594
**TAG (mg/dl)**	87.8 ± 27.55	182 ± 46.7	0.0861	129.68 ± 58.81	158 ± 63.34	0.1250

**NT2D:** *No Type 2 Diabetes***; T2D:** *Type 2 Diabetes;* **TAG**: *Triacylglycerides;* **BMI:** *Body Mass Index*. Groups were compared using a *t* test. Bold data indicate statistical significance (*p*<0.05)

**Biological validation cohort:** a cohort of 91 (55 NT2D and 36 T2D) VAT samples was obtained from patients (male and female) from the Surgery and Endocrinology Departments at the Hospital Universitario Central de Asturias (Asturias, Spain) who underwent gastric bypass. Criteria for bariatric bypass surgery were the same as the discovery cohort (data for obesity-related comorbidities: diabetes, n = 36; CVD, n = 4; non-alcoholic fatty liver disease, n = 49; and hypertension, n = 49). The anthropometric and clinical characteristics of the samples are presented in **Table 1**.

### DNA extraction and bisulfite pyrosequencing methylation analysis

Genomic DNA was isolated from VAT samples using a phenol/chloroform protocol. DNA quality control and storage was carried out as indicated in **Supplementary Methods.**

Bisulfite modification and bisulfite pyrosequencing were performed as indicated in **Supplementary Methods** Specific pyrosequencing primers were designed using Pyromark Assay Design 2.0. Software and are described in **Table 2**.

**Table 2:** Set of primers used to validate by pyrosequencing.

GENE	SEQUENCE	GenBank NUMBER	POSITION CG ANALYZED
***HOOK2*-V46**			
*BTN-FW*	5´- AGGTGGTGGGTGATATTTATATT-3´	NC_000019.10 (12765633–12766634)	12766135
*RW*	5´-CCCTAACCCTACTCTCTCCT-3		
*SEQ*	5´- CCACCTAAAAAATACCA-3´		
***HOOK2*-V78**			
*BTN -FW*	5´-TGGGAGGTGGTGGGTGGGTGAT-3´	NC_000019.10 (12765532–12766533)	12766034
*RW*	5´-AAAAAACCAAAAATAAACACAAA-3´		
*SEQ*	5´-CCAAAAATAAACACAAACACTA-3´		
***HOOK2*-V85**			
*FW*	5´-GGGAGGTGGTGGGTGATA-3´	NC_000019.10 (12765686–12766687)	12766188
*BTN-RW*	5´-CCTAACCCTACTCTCTCCT-3´		
*SEQ*	5´-GTTGGTATTTTTTAGGTGG-3´		

### Illumina 450K data methylation analysis

Methylation profiling of samples was carried out using Illumina Infinium HumanMethylation450 BeadChip Kit (Illumina Inc., USA) [19]. Data methylation analyses were performed as indicated in **S1 Supplementary Methods, S1 Fig**.

### Genomic region analysis

The probes in the microarray were assigned to a genomic region according to their position relative to the transcript information extracted from the R/Bioconductor package TxDb.Hsapiens.UCSC.hg19.knownGene [20]. Genomic region analyses were conducted as indicated in **Supplementary Methods**.

### CGI status analysis and Gene Ontology analysis

CpG island status was evaluated with respect to R. Irizarry's set of CpG island regions, included in the R/Bioconductor FDb.InfiniumMethylation.hg19 package [21, 22] and as indicated in **Supplementary Methods**.

To annotate the differentially methylated CpGs (dmCpGs) we used the HOMER annotation tool (http://homer.ucsd.edu/homer/ngs/annotation.html). All analyses were performed with a background reference comprising the whole set of genes present in the Illumina 450k platform. Results from HOMER (Hypergeometric Optimization of Motif EnRichment) were adjusted for multiple hypotheses using the Benjamini-Hochberg method for controlling the False Discovery Rate, FDR (threshold 0.05).

### Statistical analysis

In order to identify the dmCpGs, a robust moderated *t*-test (R/Bioconductor package limma, version 3.24.15) was used on every probe in the array. Resulting *p*-values were adjusted for multiple comparisons by controlling the FDR (False Discovery Rate) using the Benjamini-Hochberg method. The difference between group means was used as a measure of effect size. In addition, the Genomic Region and CpG island status analyses were performed using a standard x^2^-test, and effect size was assessed by the Odds Ratio (OR). The significance threshold was fixed at 0.05 for all analyses. All work was carried out using R/Bioconductor version 3.1. A more detailed description of each of the individual types of analysis is included in **Supplementary Methods**.

## Results

### Genome wide DNA adipocyte methylation profiling and differentially methylated genes in individuals with T2D and obesity

**Fig 1:**
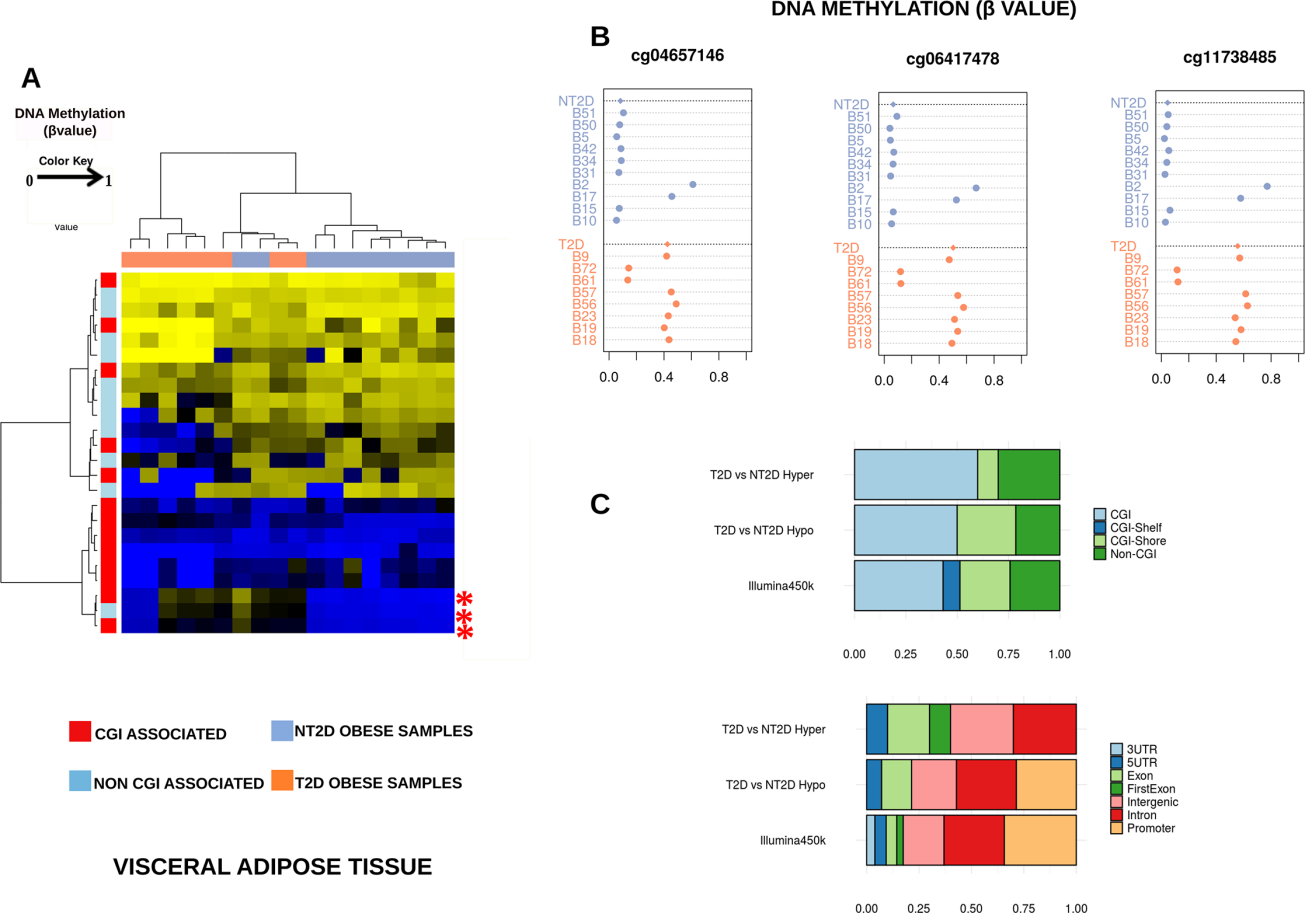
A. Hierarchical clustering heatmap. Showing differentially methylated CpGs of autosomal probes from women with obesity and discordant for type 2 diabetes (8 T2D and 10 NT2D). The heat map scale shows the range of methylation values, from 0 (blue) to 1 (yellow). Whether the CpG site analyzed is associated with a CpG island (CGI) or not can clearly be distinguished. Red asterisks mark the differentially methylated probes validated by pyrosequencing on *HOOK2* gene. B. Strip charts showing β values of three differentially methylated CpGs (dmCpGs) located on *HOOK2* gene in individual samples of the discovery cohort. The bold dotted line with the rhombus indicates the median value of each group of samples. C. Distribution of differentially methylated CpGs (dmCpGs) relative to CGIs, and relative distribution of dmCpGs across different genomic regions. *Abbreviations*: *T2D (Type 2 Diabetes); NT2D (No Type 2 Diabetes)*.

The DNA methylation status of 485,000 methylation positions was analyzed in VAT from 8 T2D, 10 NT2D (all female) samples using Illumina Infinium Methylation Arrays. Data from probes were used to calculate a β-value between 0 and 1 (equivalent to 0%-100% methylation, respectively) (Fig 1A).

We then compared the methylation (β value) of individual CpG sites in these two groups of samples. Using the robust and moderated Empirical Bayes methodology described in the Methods section, we obtained a set of 24 differentially methylated probes (adjusted *p*<0.05) (Data Accession number: https://doi.org/10.5281/zenodo.841654). Of these, 10 (related to seven genes) had a higher mean methylation value in T2D samples when compared to NT2D, Table 3. When we examined the CpG sites which were differentially methylated between T2D and NT2D samples, we found three positions (Illumina probes cg 11738485, cg 04657146 and cg 06417478) located in the intragenic region of chromosome 19, all of which overlay the same gene, *HOOK2* (Hook Microtubule Tethering Protein 2) (Δβ ≥ 0.195) (Fig 1B). In addition, 14 CpG sites (9 genes) were hypomethylated in T2D compared with NT2D samples, Table 3.

**Table 3:** Differentially methylated CpG sites in T2D compared with NT2D samples.

Ilumina probe ID	CHR	probeStart	probeEnd	CpG Location	GENE SYMBOL (1)	βT2D	SD	βNT2D	SD	Δβ (2)	Adjusted *p*Value (3)
cg13978347	chr9	120140243	120140292	Non-CGI	ASTN2	0.8316	0.2690	0.7134	0.2072	0.1181	6.8418e-08
cg11738485	chr19	12877000	12877049	CGI	HOOK2	0.4639	0.2155	0.1676	0.2714	**0.2963**	1.1924e-07
cg04657146	chr19	12876947	12876996	CGI	HOOK2	0.3638	0.1407	0.1681	0.1967	**0.1956**	4.5798e-06
cg06417478	chr19	12876798	12876847	CGI-Shore	HOOK2	0.4211	0.1885	0.1674	0.2297	**0.2537**	2.6928e-06
cg01528832	chr10	65225240	65225289	CGI	JMJD1C;JMJD1C-AS1	0.1582	0.0411	0.0807	0.0436	0.0775	7.8258e-07
cg14456004	chr13	21872349	21872398	CGI	MIPEPP3	0.3598	0.0705	0.1405	0.0984	**0.2192**	4.0028e-06
cg07234876	chr8	600039	600088	CGI	NA	0.9501	0.0658	0.8173	0.1312	0.1327	1.0884e-05
cg20991723	chr1	152506874	152506923	Non-CGI	NA	0.9133	0.0729	0.8294	0.0399	0.0838	1.9410e-06
cg01475110	chr12	90332935	90332984	Non-CGI	NA	0.9408	0.0369	0.9071	0.0182	0.0337	4.2191e-05
cg23231268	chr3	46792462	46792511	CGI	PRSS50;PRSS45	0.9744	0.0247	0.9442	0.0478	0.0302	9.4175e-06
cg00777636	chr1	6446216	6446265	Non-CGI	ACOT7	0.8602	0.0631	0.9418	0.0242	-0.0815	1.2085e-07
cg04850148	chr17	34539744	34539793	Non-CGI	CCL4L1	0.3942	0.2744	0.6513	0.1171	**-0.2570**	3.4374e-05
cg01353608	chr4	15656863	15656912	CGI	FBXL5	0.0343	0.0456	0.0783	0.1052	-0.0439	1.3711e-06
cg03635532	chr19	40376835	40376884	CGI-Shore	FCGBP	0.4121	0.4372	0.6730	0.3553	**-0.2609**	3.6600e-06
cg05073382	chr8	2045798	2045847	CGI-Shore	MYOM2	0.5436	0.3105	0.8185	0.0330	**-0.2748**	3.6176e-06
cg11066601	chr1	185373438	185373487	Non-CGI	NA	0.4611	0.1220	0.6896	0.1635	**-0.2285**	3.6065e-08
cg06675417	chr18	77292443	77292492	CGI	NA	0.3087	0.2560	0.6583	0.1063	**-0.3495**	1.3992e-06
cg03070989	chr19	34311434	34311483	CGI	NA	0.1958	0.0963	0.3195	0.1256	-0.1237	8.1823e-06
cg14168080	chr7	157504135	157504184	CGI	PTPRN2	0.3583	0.4012	0.6672	0.2257	**-0.3088**	4.2091e-06
cg11757124	chr7	157526947	157526996	CGI-Shore	PTPRN2	0.6782	0.2053	0.8522	0.0637	-0.1740	2.8778e-06
cg17729891	chr19	51107510	51107559	CGI	SNAR-F	0.1715	0.1862	0.2931	0.1452	-0.1216	9.5658e-08
cg19825302	chr19	51107512	51107561	CGI	SNAR-F	0.1780	0.2096	0.2911	0.1554	-0.1130	2.0739e-08
cg23284931	chr11	13983273	13983322	CGI-Shore	SPON1	0.7280	0.0664	0.8348	0.0394	-0.1067	1.3765e-08
cg12587985	chr7	64295583	64295632	CGI	ZNF138	0.7722	0.0613	0.8887	0.0220	-0.1165	2.6298e-07

**T2D:** Type 2 Diabetes; **NT2D:** No Type 2 Diabetes; **SD:** Standard Deviation.

(1) Gene symbol “NA” represents an intergenic region to which no known genes map.

(2) A positive value for Δbeta indicates hypermethylation in T2D in comparison to NT2D samples, and a negative value indicates hypomethylation.

(3) Resulting *p*-values were adjusted for multiple comparisons by controlling the FDR using the Benjamini-Hochberg method.

### Genomic distribution of differentially methylated CpGs in VAT samples

We compared the genomic distribution of the differentially methylated CpG sites with all the sites analyzed by the Infinium array based on the genomic location with respect to CpG islands. We did not find any significant differences, but there was an overrepresentation of CpG islands in the hypermethylated CpG sites in T2D samples with respect to NT2D samples (Pearson´s chi-squared test, adjusted *p* = 0.48; OR = 1.97). The same result was obtained with the hypomethylated CpGs in T2D, which are mainly located in CGI regions (CpG island, CpG shore (2 kb flanking the island), CpG shelf (2 kb flanking the shore)) (Pearson´s chi-squared test, adjusted *p* = 0.69; OR = 1.31) (Fig 1C).

The overall distribution of hypermethylated CpGs in relation to the nearest gene (5´UTR, 1st exon, gene body, 3´UTR) or intergenic regions in our analysis was suggestive of enrichment for the gene body (exon) in T2D compared to NT2D samples (Pearson´s chi-squared test, adjusted *p* = 0.08; OR = 4.69). In addition, hypomethylated CpG sites were over-represented in intergenic regions for T2D, although this difference did not reach significance (Pearson´s chi-squared test, adjusted *p* = 0.72; OR = 3.12) (Fig 1C).

### Biological significance of the differentially methylated genes in T2D samples

To identify the biological relevance of the differentially methylated genes between T2D and NT2D samples we performed Gene Ontology analysis. We did not find any molecular functions or biological processes that were significantly associated with the differentially methylated genes (Data not shown), although a significant enrichment in specific regions on chromosome 10 was observed, S1 Table.

### Methylation status of *HOOK2* gene in the biological validation cohort of obese samples

To confirm the results obtained from the DNA methylation array, we developed independent DNA methylation assays using bisulfite pyrosequencing. Among the genes with differential DNA methylation levels in VAT tissue from T2D individuals compared with NT2D samples we found differences in three CpGs located within the *HOOK2* gene (Δβ = 0.296; Δβ = 0.195; Δβ = 0.253).

An initial validation was made on the discovery cohort. For all CpGs analyzed, significant correlations were found between the two methods used to determine the percentage of methylation in the three probes meeting our criteria to be considered differentially methylated between T2D and NT2D (cg 04657146 probe, adjusted *p* = 1.75 x 10^−5^,R^2^ = 0.69); cg 06417478 probe, adjusted *p* = 1.95 x 10^−5^,R^2^ = 0.59; cg 11738485 probe, adjusted *p* = 3.31 x 10^−6^,R^2^ = 075), S2 Fig.

To further explore the involvement of *HOOK2* gene methylation in T2D development, bisulfite sequencing analyses were conducted in a biological validation cohort of obese samples (n = 91, 36 T2D and 55 NT2D). The degree of DNA methylation of *HOOK2* in the three regions analyzed that was observed in this way included all the differentially methylated CpGs described in the discovery cohort.

**Fig 2:**
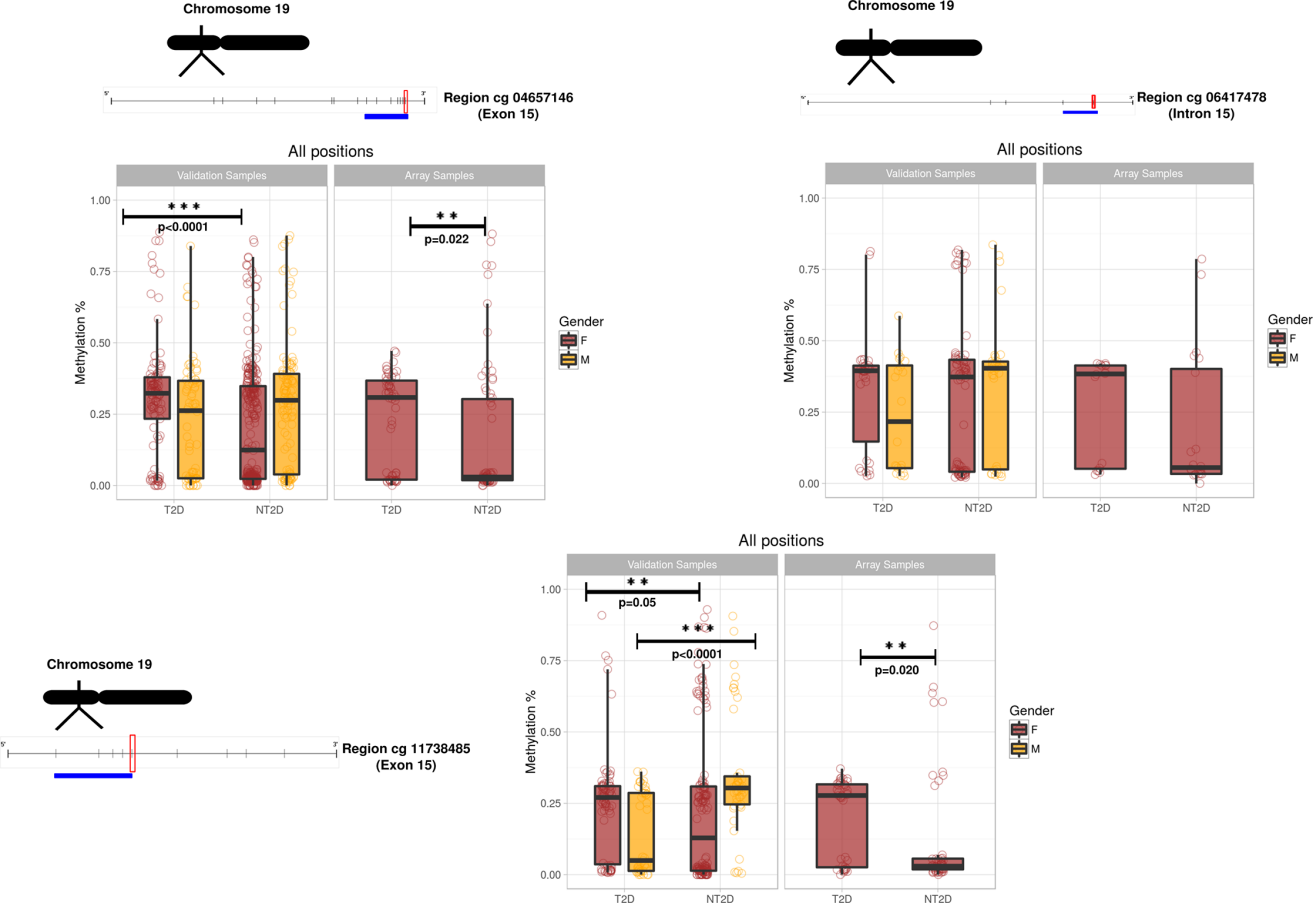
Box plots illustrate the methylation values of differentially methylated CpG regions between T2D and NT2D samples validated by pyrosequencing in *HOOK2* gene. Schematic representation of the target regions studied and the methylation values from T2D and NT2D samples in the discovery cohort and the validation cohort are shown. Vertical lines represent the location of each CpG site. The analyzed region (blue line) and the CpG sites in the array (red box) are highlighted. The *p*-values for the comparison of the groups are also indicated (**adjusted *p*-value <0.05; ***adjusted *p*-value <0.0001). *Abbreviations*: *T2D (Type 2 Diabetes); NT2D (No Type 2 Diabetes)*.

The median and the interquartile range of each CpG analyzed by pyrosequencing are shown in (Fig 2). The results demonstrate the differential methylation status of the *HOOK2* gene regions between T2D and NT2D samples. The median for the cg 04657146-region (with 7 CpG nucleotides) was significantly higher in the T2D group than the NT2D with respect to the discovery cohort (adjusted *p* = 0.022), as well as in the biological validation cohort (adjusted *p* = 0.021). Interestingly, when the validation cohort was separated by gender, the median for this region in females was significantly higher in T2D samples than the NT2D (adjusted *p* = 0.0001334), while there was no significant difference for males. In the cg 11738485-region (formed by 5 CpG nucleotides), the pattern of higher methylation in the T2D group was repeated, but only in the discovery cohort (adjusted *p* = 0.020), not in the validation cohort. However, significant differences did become apparent when the validation cohort was separated by gender, with the median in female samples being significantly higher in T2D samples than the NT2D (adjusted *p* = 0.05), while the male T2D samples were interestingly found to be significantly hypomethylated (adjusted *p* = 6.25 x 10^−5^) compared to male NT2D samples (Fig 2). In contrast, no significant results for the cg 06417478-region (formed by 2 CpG nucleotides) were obtained for either of the cohorts analyzed, even when separated by gender.

## Discussion

The prevalence of T2D is increasing worldwide and while the mechanisms responsible for increased T2D risk are still poorly understood, epigenetics is thought to be key to the process [23]. This work revealed significant changes in DNA methylation throughout the entire human genome of VAT in individuals with obesity and discordant for T2D. Significant differential methylation (BH adjusted *p*<0.05, absolute change in methylation 20%) of 10 CpG sites was observed between the two phenotypes.

In line with previously published studies [8, 24], our results for hypermethylated CpGs associated with T2D were suggestive of methylation enrichment in the gene body, though the difference did not reach significance, possibly due to the small size of the cohorts. Gene bodies are CpG-poor regions that contain multiple repetitive and transposable elements. Methylation in these regions, may function to silence repetitive DNA elements found within the gene body [24], or be responsible for changes at intron–exon boundaries, suggesting an association with the splicing that may create chimeric transcripts with dominant–negative or neomorphic effects, or through transcriptional interference and potential RNA interference (RNAi) effects if arising from antisense transcript originating at transposon promoters [25]. Recently, the work developed by Pheiffer et al [26] has postulated that the differential methylation observed in the intergenic regions of peripheral blood samples in T2D individuals is involved in microRNA regulation and in the pathogenesis of T2D development.

The list of differentially methylated genes in VAT of individuals with obesity and T2D compared with NT2D individuals in this study comprises six genes whose Δβ is greater than 0.20. These are *MIPEPP3* (Mitochondrial Intermediate Peptidase Pseudogene 3), *CCL4L1* (C-C Motif Chemokine Ligand 4 Like 1), *FCGBP* (Fc Fragment of IgG Binding Protein), *MYOM2* (Myomesin 2), *PTPRN2* (Protein Tyrosine Phosphatase, Receptor Type N2) and *HOOK2*. Among them, only *CCL4L1* could have been found to be related with insulin resistance. This gene, a CC chemokine located at chromosome 17, belongs to the same family as CCL4, which has been associated with T2D. The two proteins differ at only three amino acids and have redundant function [27]. CCL4 and its receptor CCR5 play diverse roles in the inflammatory response underlying T2D, due to their chemoattractivity towards macrophages, natural killer cells, monocytes, and immature dendritic cells, all of which have widely been described as playing a role in T2D [28]. Increased serum levels of CCL4 produced by islet cells have also been reported in type 1 diabetes and pre-diabetic condition, as well as in patients with T2D, suggesting the involvement of CCL4 in various stages of this disease [29,30]. The role of CCL4L1 in T2D has not been described, but the hypomethylation observed in VAT of individuals with T2D, is thought to perhaps contribute to the inflammatory response associated with insulin resistance in adipose tissue. *PTPRN2* has been identified as an autoantigen in type 1 diabetes [31]. As a transmembrane protein, PTPRN2 shuttles between secretory vesicles and the plasma membrane, and has been implicated in insulin exocytosis [32]. However, its precise role in the secretory pathway is unknown. Differential *PTPRN2* methylation has been described in placental samples of newborns with intrauterine growth restricted (IUGR) conditions [33], a condition that has been linked to the development of T2D later in life [34].

Extending the list of genes linked to T2D, this work provides evidence to support the inclusion of the gene *HOOK2*. We have found aberrant intragenic DNA methylation of this gene in a population with obesity and T2D when compared with a group of people with obesity but without T2D. Differential methylation status was found in all the HOOK2 gene regions validated by pyrosequencing, and, while low sample number is a potential limitation, these differences were significant with respect to two of the three regions. However, to separate the effects of HOOK2 methylation and the numerous confounding factors, we will need much larger numbers of samples and more detailed clinical information. Only through meta-analyses combining genome-wide data sets generated in different laboratories with different cohorts will we be able to reveal a more complete picture. Another potential limitation of this work is the low methylation difference we have found (close to 20%). Although previous reports indicate that methylation differences over 10% in Illumina assays reach biological significance and have a low probability of being technical artifacts [35, 36], this remains theoretical, and the work here, unfortunately, does not demonstrate the functional significance of *HOOK2* methylation in patients with T2D. Another issue is the lack of quantification in our study of other C modifications, such as 5hmC (5-hydroxymethylcytosine), that have been reported to be associated with demethylation processes [37], regulation of gene expression [38], and changes in the chromatin structure [39] which may also show differences between the populations analyzed and provide another piece of the puzzle of our knowledge of T2D.

The significant hypermethylation observed on the cg04657146-region in T2D female samples indicates it may be a key region of the *HOOK2* gene for DNA modification potentially involved in attendant T2D in individuals with obesity, especially females. However, additional studies are required to identify the role of DNA methylation in the cg04657146-region with respect to transcription factor recognition, chromatin remodeling and *HOOK2* gene expression. In addition, and strikingly, the cg 11738485-region was found hypermethylated in T2D female samples, but hypomethylated in T2D male samples. Similar differences were observed in the work developed by Hall et al [40] who, in a genome wide DNA methylation analysis conducted on human pancreatic islets, found sex-specific differences in the DNA methylation levels of several genes located in autosomal chromosomes. The fact that these sex-biased genes involved in the functioning of pancreatic islets and insulin pathways showed differential expression could be related to the phenotypic differences observed between males and females in relation to insulin response [41, 42].

Adipose tissue function and distribution can be regulated by sexually dimorphic genes, some of which are present in VAT and controlled by sex hormones [43, 44]. From childhood on, fat distribution is different in males and females, and the difference becomes more marked during adolescence and remains this way until the menopause, when the reduction in estrogens levels means that fat distribution in women acquires an android distribution. Possibly, exposure to female sex hormones within the context of a fat and glucose rich diet, which leads to an abnormal accumulation of VAT (central adiposity), could go some way to explaining the differences observed in the methylation the HOOK2 gene in men and women with T2D. However, larger study groups, preferably with longitudinal sample collection of HOOK2 gene methylation would be needed to confirm the sexual differences observed in the methylation of this gene and its contribution to the different strategies for glycemic maintenance adopted by males and females.

The role of HOOK2 in primary cilia assembly has been described [45]. Primary cilia project out from the cellular membrane in most vertebrate cells and exert a sensor function in relation to stimuli (light, chemical and mechanical), triggering signaling pathways that maintain cell homeostasis [46]. Defects in this structure lead to a group of disorders called ciliopathies, two of which, the Bardet-Biedl and Alström syndromes, count T2D among their clinical manifestations [47]. Given that T2D and obesity are common features in ciliopathies and that HOOK2 is involved in primary cilia structure; the *HOOK2* hypermethylation observed in the T2D samples in this work, would suggest that the possible role indicated here for this protein in T2D development may be mediated through alterations to primary cilia.

Another possibility is that HOOK2 protein could be involved in GLUT4 (glucose transporter type 4) traffic. The HOOK family is a group of cytoplasmic linker proteins associated with microtubules that participate in microtubule-vesicle transport and organelle support. Microtubules play a central role in GLUT4 translocation and glucose uptake [48–51]. The role of this microtubule-associated protein in insulin secretion or GLUT-4 traffic is still unclear, but *HOOK2* methylation observed in T2D samples in the current work could perhaps be altering its function within the adipocyte. Benton et al [52], analyzing genome wide DNA methylation before and after bariatric surgery, suggested a similar role for the *MYO1C* (Myosin IC) gene, which codifies for an actin-related protein involved in GLUT4 translocation.

In conclusion, the differential methylation profiling we have described in individuals with T2D and obesity, and, more specifically, the methylation differences observed on the *HOOK2* gene, points to the possible contribution of epigenetic factors, along with others previously described, to T2D predisposition. However, supplementary studies are required to identify the exact role of HOOK2 protein in the development of this pathology, as well as in terms of its potential role as a biomarker of increased risk, or in the design of gender-specific antidiabetic treatments.

## Supporting information

S1 Fig Experimental workflow of Illumina 450K data methylation analysis. (TIF)

S2 Fig Correlations between Illumina 450 K array data and pyrosequencing analysis on *HOOK2* gene in the discovery cohort. Representative data for a single CpG site for the three probes analyzed are shown. Illumina probe IDs are indicated after gene name.
(TIF)

S1 Table Gene Ontology of hypermethylated genes in individuals with T2D compared with NT2D. (DOC)

S1 File Supplementary methods. (DOC)

## References

[1] Alvarez-Castro P, Sangiao-Alvarellos S, Brandon-Sanda I, Cordido F. [Endocrine function in obesity]. Endocrinol Nutr [Internet]. 2011/08/10. 2011;58(8):422–32. Available from: http://www.ncbi.nlm.nih.gov/entrez/query.fcgi?cmd=Retrieve&db=PubMed&dopt=Citation&list_uids=21824829 doi: 10.1016/j.endonu.2011.05.015 21824829 10.1016/j.endonu.2011.05.015

[2] Ros Perez M, Medina-Gomez G. [Obesity, adipogenesis and insulin resistance]. Endocrinol Nutr [Internet]. 2011/07/23. 2011;58(7):360–9. Available from: http://www.ncbi.nlm.nih.gov/entrez/query.fcgi?cmd=Retrieve&db=PubMed&dopt=Citation&list_uids=21778123 doi: 10.1016/j.endonu.2011.05.008 21778123 10.1016/j.endonu.2011.05.008

[3] Xu L, Kitade H, Ni Y, Ota T. Roles of Chemokines and Chemokine Receptors in Obesity-Associated Insulin Resistance and Nonalcoholic Fatty Liver Disease. Biomolecules [Internet]. 2015/07/22. 2015;5(3):1563–79. Available from: http://www.ncbi.nlm.nih.gov/entrez/query.fcgi?cmd=Retrieve&db=PubMed&dopt=Citation&list_uids=26197341 doi: 10.3390/biom5031563 26197341 10.3390/biom5031563 PMC4598764

[4] McCarthy MI. Genomics, Type 2 Diabetes, and Obesity. Feero WG, Guttmacher AE, editors. N Engl J Med [Internet]. 2010 12 9 [cited 2017 Aug 10];363(24):2339–50. Available from: http://www.ncbi.nlm.nih.gov/pubmed/21142536 doi: 10.1056/NEJMra0906948 21142536 10.1056/NEJMra0906948

[5] Drong AW, Lindgren CM, McCarthy MI. The genetic and epigenetic basis of type 2 diabetes and obesity. Clin Pharmacol Ther [Internet]. 2012 12 10 [cited 2017 Aug 10];92(6):707–15. Available from: http://doi.wiley.com/10.1038/clpt.2012.149 23047653 10.1038/clpt.2012.149 PMC7116747

[6] Rönn T, Volkov P, Davegårdh C, Dayeh T, Hall E, Olsson AH, et al A six months exercise intervention influences the genome-wide DNA methylation pattern in human adipose tissue. Greally JM, editor. PLoS Genet [Internet]. 2013 6 27 [cited 2017 Aug 10];9(6):e1003572 Available from: http://dx.plos.org/10.1371/journal.pgen.1003572 doi: 10.1371/journal.pgen.1003572 23825961 10.1371/journal.pgen.1003572 PMC3694844

[7] Ribel-Madsen R, Fraga MF, Jacobsen S, Bork-Jensen J, Lara E, Calvanese V, et al Genome-wide analysis of DNA methylation differences in muscle and fat from monozygotic twins discordant for type 2 diabetes. Dahlman IA, editor. PLoS One [Internet]. 2012 12 10 [cited 2017 Aug 10];7(12):e51302 Available from: http://dx.plos.org/10.1371/journal.pone.0051302 doi: 10.1371/journal.pone.0051302 23251491 10.1371/journal.pone.0051302 PMC3519577

[8] Nilsson E, Jansson PA, Perfilyev A, Volkov P, Pedersen M, Svensson MK, et al Altered DNA methylation and differential expression of genes influencing metabolism and inflammation in adipose tissue from subjects with type 2 diabetes. Diabetes [Internet]. 2014 9 1 [cited 2017 Aug 10];63(9):2962–76. Available from: http://diabetes.diabetesjournals.org/cgi/doi/10.2337/db13-1459 24812430 10.2337/db13-1459

[9] Chen Y-T, Liao J-W, Tsai Y-C, Tsai F-J. Inhibition of DNA methyltransferase 1 increases nuclear receptor subfamily 4 group A member 1 expression and decreases blood glucose in type 2 diabetes. Oncotarget [Internet]. 2016 6 28 [cited 2017 Aug 10];7(26):39162–70. Available from: http://www.oncotarget.com/fulltext/10043 doi: 10.18632/oncotarget.10043 27322146 10.18632/oncotarget.10043 PMC5129922

[10] Volkov P, Olsson AH, Gillberg L, Jorgensen SW, Brons C, Eriksson KF, et al A Genome-Wide mQTL Analysis in Human Adipose Tissue Identifies Genetic Variants Associated with DNA Methylation, Gene Expression and Metabolic Traits. PLoS One [Internet]. 2016/06/21. 2016;11(6):e0157776 Available from: http://www.ncbi.nlm.nih.gov/entrez/query.fcgi?cmd=Retrieve&db=PubMed&dopt=Citation&list_uids=27322064 doi: 10.1371/journal.pone.0157776 27322064 10.1371/journal.pone.0157776 PMC4913906

[11] Matsuzawa Y, Shimomura I, Nakamura T, Keno Y, Kotani K, Tokunaga K. Pathophysiology and pathogenesis of visceral fat obesity. Obes Res [Internet]. 1995 9 [cited 2017 Aug 10];3 Suppl 2:187S–194S. Available from: http://www.ncbi.nlm.nih.gov/pubmed/8581775 8581775 10.1002/j.1550-8528.1995.tb00462.x

[12] Garg A. Regional adiposity and insulin resistance. J Clin Endocrinol Metab [Internet]. 2004/09/10. 2004;89(9):4206–10. Available from: http://www.ncbi.nlm.nih.gov/entrez/query.fcgi?cmd=Retrieve&db=PubMed&dopt=Citation&list_uids=15356007 doi: 10.1210/jc.2004-0631 15356007 10.1210/jc.2004-0631

[13] Preis SR, Massaro JM, Robins SJ, Hoffmann U, Vasan RS, Irlbeck T, et al Abdominal subcutaneous and visceral adipose tissue and insulin resistance in the Framingham heart study. Obesity (Silver Spring) [Internet]. 2010 11 25 [cited 2017 Aug 10];18(11):2191–8. Available from: http://doi.wiley.com/10.1038/oby.2010.59 20339361 10.1038/oby.2010.59 PMC3033570

[14] Arner P, Sinha I, Thorell A, Ryden M, Dahlman-Wright K, Dahlman I. The epigenetic signature of subcutaneous fat cells is linked to altered expression of genes implicated in lipid metabolism in obese women. Clin Epigenetics [Internet]. 2015/09/10. 2015;7(1):93 Available from: http://www.ncbi.nlm.nih.gov/entrez/query.fcgi?cmd=Retrieve&db=PubMed&dopt=Citation&list_uids=26351548 26351548 10.1186/s13148-015-0126-9 PMC4562340

[15] Crujeiras AB, Diaz-Lagares A, Moreno-Navarrete JM, Sandoval J, Hervas D, Gomez A, et al Genome-wide DNA methylation pattern in visceral adipose tissue differentiates insulin-resistant from insulin-sensitive obese subjects. Transl Res [Internet]. 2016 12 [cited 2017 Aug 10];178:13–24.e5. Available from: http://linkinghub.elsevier.com/retrieve/pii/S1931524416301074 doi: 10.1016/j.trsl.2016.07.002 27477082 10.1016/j.trsl.2016.07.002

[16] Keller M, Kralisch S, Rohde K, Schleinitz D, Dietrich A, Schön MR, et al Global DNA methylation levels in human adipose tissue are related to fat distribution and glucose homeostasis. Diabetologia [Internet]. 2014 11 22 [cited 2017 Aug 10];57(11):2374–83. Available from: http://www.ncbi.nlm.nih.gov/pubmed/25145546 doi: 10.1007/s00125-014-3356-z 25145546 10.1007/s00125-014-3356-z

[17] Keller M, Hopp L, Liu X, Wohland T, Rohde K, Cancello R, et al Genome-wide DNA promoter methylation and transcriptome analysis in human adipose tissue unravels novel candidate genes for obesity. Mol Metab [Internet]. 2017 1 [cited 2017 Aug 10];6(1):86–100. Available from: http://linkinghub.elsevier.com/retrieve/pii/S2212877816302757 doi: 10.1016/j.molmet.2016.11.003 28123940 10.1016/j.molmet.2016.11.003 PMC5220399

[18] Guénard F, Tchernof A, Deshaies Y, Pérusse L, Biron S, Lescelleur O, et al Differential methylation in visceral adipose tissue of obese men discordant for metabolic disturbances. Physiol Genomics [Internet]. 2014 3 15 [cited 2017 Aug 10];46(6):216–22. Available from: doi: 10.1152/physiolgenomics.00160.2013 24495915 10.1152/physiolgenomics.00160.2013

[19] Sandoval J, Heyn H, Moran S, Serra-Musach J, Pujana MA, Bibikova M, et al Validation of a DNA methylation microarray for 450,000 CpG sites in the human genome. Epigenetics [Internet]. 2011/05/20. 2011;6(6):692–702. Available from: http://www.ncbi.nlm.nih.gov/entrez/query.fcgi?cmd=Retrieve&db=PubMed&dopt=Citation&list_uids=21593595 21593595 10.4161/epi.6.6.16196

[20] Carlson M. TxDb.Hsapiens.UCSC.hg19.knownGene: Annotation for TranscriptDb object(s). R package version 2.9.2.

[21] Triche TJ. FDb.InfiniumMethylation.hg19: Annotation package for Illumina Infinium DNA methylation array probes. R package version 1.0.1.

[22] Wu H, Caffo B, Jaffee HA, Irizarry RA, Feinberg AP. Redefining CpG islands using hidden Markov models. Biostatistics [Internet]. 2010/3/10 11(3):499–514. Available from: http://www.ncbi.nlm.nih.gov/entrez/query.fcgi?cmd=Retrieve&db=PubMed&dopt=Citation&list_uids=20212320 doi: 10.1093/biostatistics/kxq005 20212320 10.1093/biostatistics/kxq005 PMC2883304

[23] Ling C, Rönn T. Epigenetic markers to further understand insulin resistance. Diabetologia [Internet]. 2016 11 20 [cited 2017 Aug 10];59(11):2295–7. Available from: http://link.springer.com/10.1007/s00125-016-4109-y doi: 10.1007/s00125-016-4109-y 27650286 10.1007/s00125-016-4109-y

[24] Yoder JA, Walsh CP, Bestor TH. Cytosine methylation and the ecology of intragenomic parasites. Trends Genet [Internet]. 1997 8 [cited 2017 Aug 29];13(8):335–40. Available from: http://www.ncbi.nlm.nih.gov/pubmed/9260521 9260521 10.1016/s0168-9525(97)01181-5

[25] Laurent L, Wong E, Li G, Huynh T, Tsirigos A, Ong CT, et al Dynamic changes in the human methylome during differentiation. Genome Res [Internet]. 2010 3 1 [cited 2017 Aug 29];20(3):320–31. Available from: http://www.ncbi.nlm.nih.gov/pubmed/20133333 doi: 10.1101/gr.101907.109 20133333 10.1101/gr.101907.109 PMC2840979

[26] Pheiffer C, Erasmus RT, Kengne AP, Matsha TE. Differential DNA methylation of microRNAs within promoters, intergenic and intragenic regions of type 2 diabetic, pre-diabetic and non-diabetic individuals. Clin Biochem [Internet]. 2015/12/15. 2015;pii: S0009-9120(15)00552-4. Available from: http://www.ncbi.nlm.nih.gov/entrez/query.fcgi?cmd=Retrieve&db=PubMed&dopt=Citation&list_uids=26656639 10.1016/j.clinbiochem.2015.11.021 26656639

[27] Howard OMZ, Turpin JA, Goldman R, Modi WS. Functional redundancy of the human CCL4 and CCL4L1 chemokine genes. Biochem Biophys Res Commun [Internet]. 2004 7 30 [cited 2017 Aug 10];320(3):927–31. Available from: http://linkinghub.elsevier.com/retrieve/pii/S0006291X04012689 doi: 10.1016/j.bbrc.2004.06.039 15240137 10.1016/j.bbrc.2004.06.039

[28] Lackey DE, Olefsky JM. Regulation of metabolism by the innate immune system. Nat Rev Endocrinol [Internet]. 2016 1 10 [cited 2017 Aug 10];12(1):15–28. Available from: doi: 10.1038/nrendo.2015.189 26553134 10.1038/nrendo.2015.189

[29] Chang T-T, Chen J-W. Emerging role of chemokine CC motif ligand 4 related mechanisms in diabetes mellitus and cardiovascular disease: friends or foes? Cardiovasc Diabetol [Internet]. 2016 12 24 [cited 2017 Aug 10];15(1):117 Available from: http://cardiab.biomedcentral.com/articles/10.1186/s12933-016-0439-9 doi: 10.1186/s12933-016-0439-9 27553774 10.1186/s12933-016-0439-9 PMC4995753

[30] Pham MN, Hawa MI, Roden M, Schernthaner G, Pozzilli P, Buzzetti R, et al Increased serum concentrations of adhesion molecules but not of chemokines in patients with Type 2 diabetes compared with patients with Type 1 diabetes and latent autoimmune diabetes in adult age: action LADA 5. Diabet Med [Internet]. 2012 4 [cited 2017 Aug 10];29(4):470–8. Available from: doi: 10.1111/j.1464-5491.2011.03546.x 22150724 10.1111/j.1464-5491.2011.03546.x

[31] Bason C, Lorini R, Lunardi C, Dolcino M, Giannattasio A, d’Annunzio G, et al In type 1 diabetes a subset of anti-coxsackievirus B4 antibodies recognize autoantigens and induce apoptosis of pancreatic beta cells. von Herrath MG, editor. PLoS One [Internet]. 2013 2 28 [cited 2017 Aug 10];8(2):e57729 Available from: http://dx.plos.org/10.1371/journal.pone.0057729 doi: 10.1371/journal.pone.0057729 23469060 10.1371/journal.pone.0057729 PMC3585221

[32] Cai T, Hirai H, Zhang G, Zhang M, Takahashi N, Kasai H, et al Deletion of Ia-2 and/or Ia-2β in mice decreases insulin secretion by reducing the number of dense core vesicles. Diabetologia [Internet]. 2011 9 6 [cited 2017 Aug 10];54(9):2347–57. Available from: http://link.springer.com/10.1007/s00125-011-2221-6 doi: 10.1007/s00125-011-2221-6 21732083 10.1007/s00125-011-2221-6 PMC3168514

[33] Chen P-Y, Chu A, Liao W-W, Rubbi L, Janzen C, Hsu F-M, et al Prenatal Growth Patterns and Birthweight Are Associated With Differential DNA Methylation and Gene Expression of Cardiometabolic Risk Genes in Human Placentas: A Discovery-Based Approach. Reprod Sci [Internet]. 2017 1 1 [cited 2017 Aug 10];1933719117716779. Available from: http://www.ncbi.nlm.nih.gov/pubmed/28693373 10.1177/1933719117716779 PMC6348426 28693373

[34] Pinney SE. Intrauterine Growth Retardation—A Developmental Model of Type 2 Diabetes. Drug Discov Today Dis Models [Internet]. NIH Public Access; 2013 [cited 2017 Aug 29];10(2):e71–7. Available from: http://www.ncbi.nlm.nih.gov/pubmed/24949076 doi: 10.1016/j.ddmod.2013.01.003 24949076 10.1016/j.ddmod.2013.01.003 PMC4058781

[35] Yuen RK, Peñaherrera MS, Dadelszen P von, McFadden DE, Robinson WP. DNA methylation profiling of human placentas reveals promoter hypomethylation of multiple genes in early-onset preeclampsia. Eur J Hum Genet [Internet]. Nature Publishing Group; 2010 [cited 2017 Aug 29];18(9):1006 Available from: https://www.ncbi.nlm.nih.gov/pmc/articles/PMC2987406/ doi: 10.1038/ejhg.2010.63 20442742 10.1038/ejhg.2010.63 PMC2987406

[36] Nakamura K, Aizawa K, Nakabayashi K, Kato N, Yamauchi J, Hata K, et al DNA methyltransferase inhibitor zebularine inhibits human hepatic carcinoma cells proliferation and induces apoptosis. PLoS One [Internet]. Public Library of Science; 2013 [cited 2017 Aug 29];8(1):e54036 Available from: http://www.ncbi.nlm.nih.gov/pubmed/23320119 doi: 10.1371/journal.pone.0054036 23320119 10.1371/journal.pone.0054036 PMC3540068

[37] Bachman M, Uribe-Lewis S, Yang X, Williams M, Murrell A, Balasubramanian S. 5-Hydroxymethylcytosine is a predominantly stable DNA modification. Nat Chem [Internet]. 2014 9 21 [cited 2017 Aug 29];6(12):1049–55. Available from: http://www.ncbi.nlm.nih.gov/pubmed/25411882 doi: 10.1038/nchem.2064 25411882 10.1038/nchem.2064 PMC4382525

[38] Branco MR, Ficz G, Reik W. Uncovering the role of 5-hydroxymethylcytosine in the epigenome. Nat Rev Genet [Internet]. 2011 11 15 [cited 2017 Aug 30];13(1):7–13. Available from: http://www.nature.com/doifinder/10.1038/nrg3080 22083101 10.1038/nrg3080

[39] Valinluck V, Tsai H-H, Rogstad DK, Burdzy A, Bird A, Sowers LC. Oxidative damage to methyl-CpG sequences inhibits the binding of the methyl-CpG binding domain (MBD) of methyl-CpG binding protein 2 (MeCP2). Nucleic Acids Res [Internet]. Oxford University Press; 2004 [cited 2017 Aug 30];32(14):4100–8. Available from: http://www.ncbi.nlm.nih.gov/pubmed/15302911 doi: 10.1093/nar/gkh739 15302911 10.1093/nar/gkh739 PMC514367

[40] Hall E, Volkov P, Dayeh T, Esguerra JL, Salo S, Eliasson L, et al Sex differences in the genome-wide DNA methylation pattern and impact on gene expression, microRNA levels and insulin secretion in human pancreatic islets. Genome Biol [Internet]. 2014/12/18. 2014;15(12):522 Available from: http://www.ncbi.nlm.nih.gov/entrez/query.fcgi?cmd=Retrieve&db=PubMed&dopt=Citation&list_uids=25517766 doi: 10.1186/s13059-014-0522-z 25517766 10.1186/s13059-014-0522-z PMC4256841

[41] Garcia-Carrizo F, Priego T, Szostaczuk N, Palou A, Picó C. Sexual Dimorphism in the Age-Induced Insulin Resistance, Liver Steatosis, and Adipose Tissue Function in Rats. Front Physiol [Internet]. Frontiers Media SA; 2017 [cited 2017 Aug 30];8:445 Available from: http://www.ncbi.nlm.nih.gov/pubmed/28744221 doi: 10.3389/fphys.2017.00445 28744221 10.3389/fphys.2017.00445 PMC5504177

[42] Kautzky-Willer A, Brazzale AR, Moro E, Vrbikova J, Bendlova B, Sbrignadello S, et al Influence of increasing BMI on insulin sensitivity and secretion in normotolerant men and women of a wide age span. Obes (Silver Spring) [Internet]. 2012/01/28. 2012;20(10):1966–73. Available from: http://www.ncbi.nlm.nih.gov/entrez/query.fcgi?cmd=Retrieve&db=PubMed&dopt=Citation&list_uids=22282046 10.1038/oby.2011.384 22282046

[43] Min JL, Nicholson G, Halgrimsdottir I, Almstrup K, Petri A, Barrett A, et al Coexpression network analysis in abdominal and gluteal adipose tissue reveals regulatory genetic loci for metabolic syndrome and related phenotypes. PLoS Genet [Internet]. 2012/03/03. 2012;8(2):e1002505 Available from: http://www.ncbi.nlm.nih.gov/entrez/query.fcgi?cmd=Retrieve&db=PubMed&dopt=Citation&list_uids=22383892 doi: 10.1371/journal.pgen.1002505 22383892 10.1371/journal.pgen.1002505 PMC3285582

[44] van Nas A, Guhathakurta D, Wang SS, Yehya N, Horvath S, Zhang B, et al Elucidating the role of gonadal hormones in sexually dimorphic gene coexpression networks. Endocrinology [Internet]. 2008/11/01. 2009;150(3):1235–49. Available from: http://www.ncbi.nlm.nih.gov/entrez/query.fcgi?cmd=Retrieve&db=PubMed&dopt=Citation&list_uids=18974276 doi: 10.1210/en.2008-0563 18974276 10.1210/en.2008-0563 PMC2654741

[45] Baron Gaillard CL, Pallesi-Pocachard E, Massey-Harroche D, Richard F, Arsanto JP, Chauvin JP, et al Hook2 is involved in the morphogenesis of the primary cilium. Mol Biol Cell [Internet]. 2011/10/15. 2011;22(23):4549–62. Available from: http://www.ncbi.nlm.nih.gov/entrez/query.fcgi?cmd=Retrieve&db=PubMed&dopt=Citation&list_uids=21998199 doi: 10.1091/mbc.E11-05-0405 21998199 10.1091/mbc.E11-05-0405 PMC3226474

[46] Oh EC, Vasanth S, Katsanis N. Metabolic regulation and energy homeostasis through the primary Cilium. Cell Metab [Internet]. 2014/12/30. 2015;21(1):21–31. Available from: http://www.ncbi.nlm.nih.gov/entrez/query.fcgi?cmd=Retrieve&db=PubMed&dopt=Citation&list_uids=25543293 doi: 10.1016/j.cmet.2014.11.019 25543293 10.1016/j.cmet.2014.11.019 PMC4370781

[47] Waters AM, Beales PL. Ciliopathies: an expanding disease spectrum. Pediatr Nephrol [Internet]. 2011/01/07. 2011;26(7):1039–56. Available from: http://www.ncbi.nlm.nih.gov/entrez/query.fcgi?cmd=Retrieve&db=PubMed&dopt=Citation&list_uids=21210154 doi: 10.1007/s00467-010-1731-7 21210154 10.1007/s00467-010-1731-7 PMC3098370

[48] Fletcher LM, Welsh GI, Oatey PB, Tavare JM. Role for the microtubule cytoskeleton in GLUT4 vesicle trafficking and in the regulation of insulin-stimulated glucose uptake. Biochem J [Internet]. 2000/11/22. 2000;352 Pt 2:267–76. Available from: http://www.ncbi.nlm.nih.gov/entrez/query.fcgi?cmd=Retrieve&db=PubMed&dopt=Citation&list_uids=11085918 11085918 PMC1221456

[49] Liu LZ, Cheung SC, Lan LL, Ho SK, Chan JC, Tong PC. Microtubule network is required for insulin-induced signal transduction and actin remodeling. Mol Cell Endocrinol [Internet]. 2012/09/22. 2013;365(1):64–74. Available from: http://www.ncbi.nlm.nih.gov/entrez/query.fcgi?cmd=Retrieve&db=PubMed&dopt=Citation&list_uids=22996137 doi: 10.1016/j.mce.2012.09.005 22996137 10.1016/j.mce.2012.09.005

[50] Muretta JM, Mastick CC. How insulin regulates glucose transport in adipocytes. Vitam Horm [Internet]. 2009/03/03. 2009;80:245–86. Available from: http://www.ncbi.nlm.nih.gov/entrez/query.fcgi?cmd=Retrieve&db=PubMed&dopt=Citation&list_uids=19251041 doi: 10.1016/S0083-6729(08)00610-9 19251041 10.1016/S0083-6729(08)00610-9

[51] Dawicki-McKenna JM, Goldman YE, Ostap EM. Sites of glucose transporter-4 vesicle fusion with the plasma membrane correlate spatially with microtubules. PLoS One [Internet]. 2012/08/24. 2012;7(8):e43662 Available from: http://www.ncbi.nlm.nih.gov/entrez/query.fcgi?cmd=Retrieve&db=PubMed&dopt=Citation&list_uids=22916292 doi: 10.1371/journal.pone.0043662 22916292 10.1371/journal.pone.0043662 PMC3423385

[52] Benton MC, Johnstone A, Eccles D, Harmon B, Hayes MT, Lea RA, et al An analysis of DNA methylation in human adipose tissue reveals differential modification of obesity genes before and after gastric bypass and weight loss. Genome Biol [Internet]. 2015/02/05. 2015;16:8 Available from: http://www.ncbi.nlm.nih.gov/entrez/query.fcgi?cmd=Retrieve&db=PubMed&dopt=Citation&list_uids=25651499 doi: 10.1186/s13059-014-0569-x 25651499 10.1186/s13059-014-0569-x PMC4301800

