# Supplementary figures and images for: Altered intragenic DNA methylation of HOOK2 gene in adipose tissue from individuals with obesity and type 2 diabetes
Source: PLoS One. 2017 Dec 11;12(12):e0189153. doi: 10.1371/journal.pone.0189153 (PMC5724849; doi:10.1371/journal.pone.0189153)

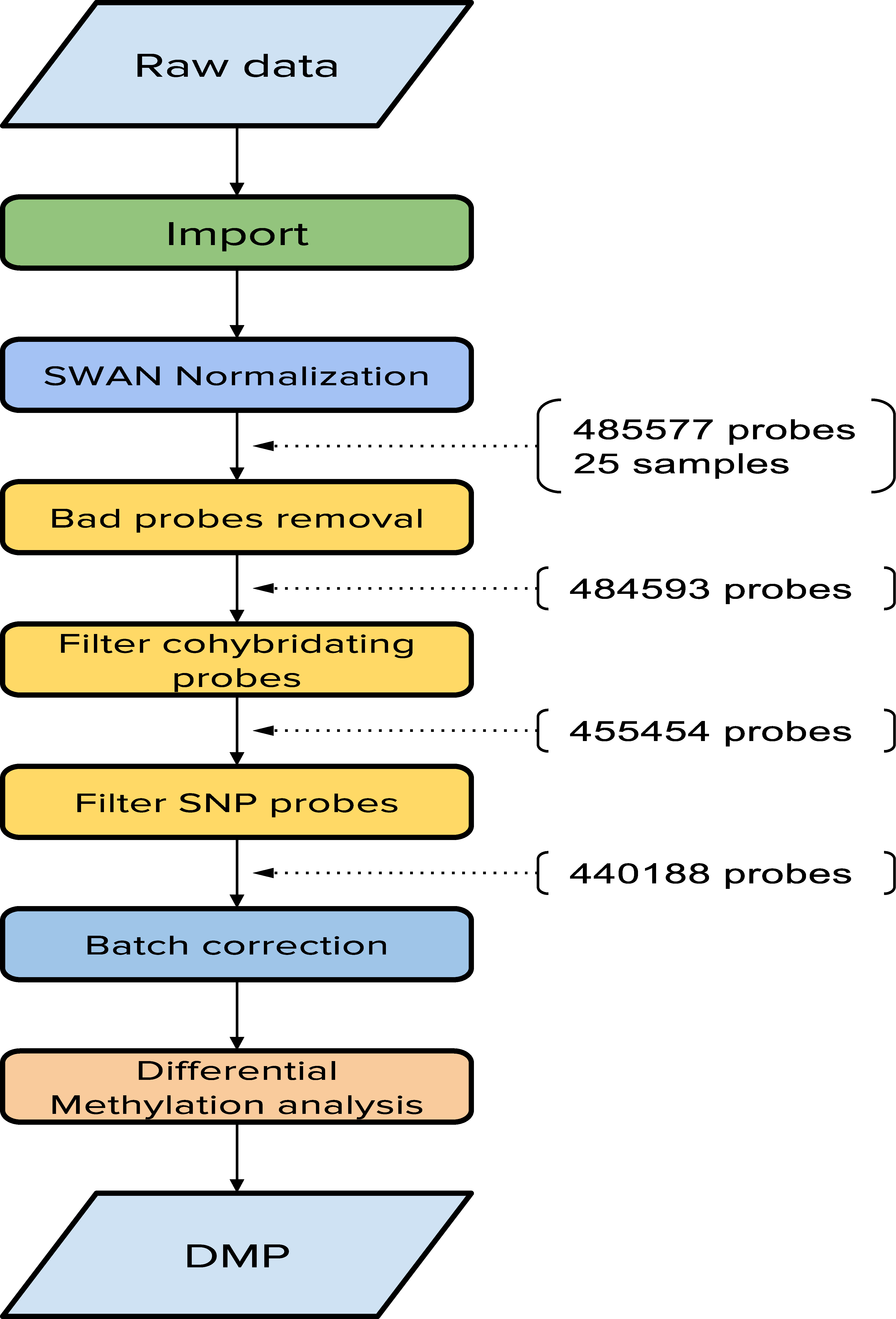

Supplement: S1 Fig — (TIF) [file pone.0189153.s001.tif]

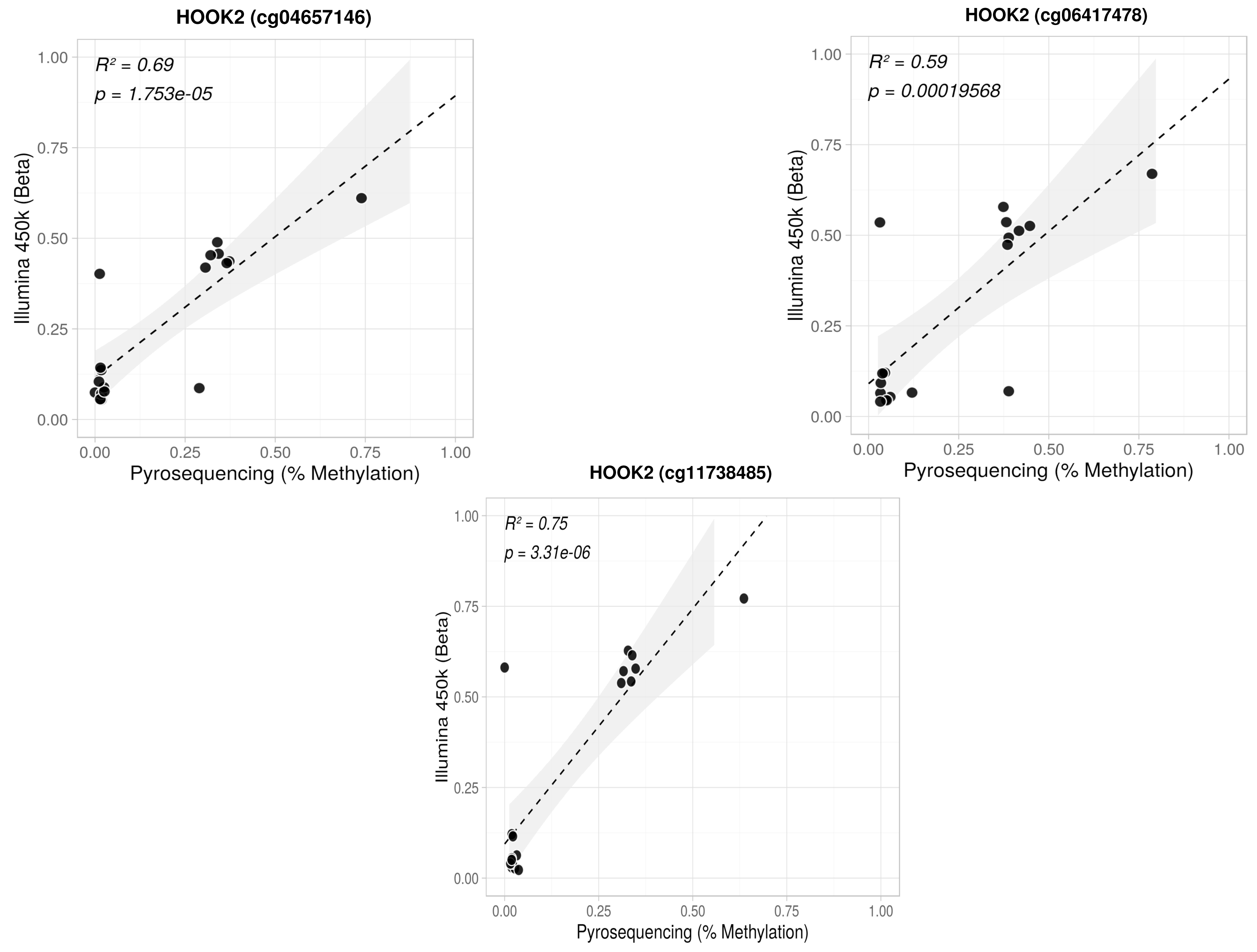

Supplement: S2 Fig — Representative data for a single CpG site for the three probes analyzed are shown. Illumina probe IDs are indicated after gene name. (TIF) [file pone.0189153.s002.tif]
