# Supplementary material for: Altered intragenic DNA methylation of HOOK2 gene in adipose tissue from individuals with obesity and type 2 diabetes
Source: PLoS One. 2017 Dec 11;12(12):e0189153. doi: 10.1371/journal.pone.0189153 (PMC5724849; doi:10.1371/journal.pone.0189153)
Supplement: S1 Table — (DOC) [file pone.0189153.s003.doc]

**Supplementary Table S1. Gene Ontology of hypermethylated genes in T2D samples compared with NT2D**

| **Onthology** | **TermID** | **Term** | **Target_Genes_In_Term** | **Genes_In_Term** | **Total_Target_Genes** | **Total_Genes** | **P_Value** | **RR** | **RR_CI_low** | **RR_CI_high** | **Log_2_RR** | **Q_Value** | **Log_10_Q_Value** |
| --- | --- | --- | --- | --- | --- | --- | --- | --- | --- | --- | --- | --- | --- |
| chromosome | human chr10q21.3 | human chr10q21.3 | 2 | 16 | 7 | 22338 | 1.0079e-05 | 455735 | 95.30 | 2179.37 | 8.83 | 0.02 | -1.69 |
