# Supplementary material for: Altered intragenic DNA methylation of HOOK2 gene in adipose tissue from individuals with obesity and type 2 diabetes
Source: PLoS One. 2017 Dec 11;12(12):e0189153. doi: 10.1371/journal.pone.0189153 (PMC5724849; doi:10.1371/journal.pone.0189153)
Supplement: S1 File — (DOC) [file pone.0189153.s004.doc]

**Methods Supplementary Information**

**Methods Section**

**DNA extraction and bisulfite pyrosequencing methylation analysis**

Genomic DNA was isolated from VAT samples using a phenol/chloroform protocol. Samples were homogenized in buffer (0.1 M NaCl, 20 mM Tris pH8, 25 mM EDTA, 0.5% SDS) and proteinase K for DNA extraction. After two incubations with phenol/chloroform/isoamyl alcohol (25:24:1), the DNA was precipitated with isopropanol, and, after washing in 70% ethanol, was resuspended in sterile milliQ water and stored at -80 ºC until use.

The DNA purity and quality was determined by agarose gel electrophoresis and confirmed by A260/280 ratio >1.8 on a Nanodrop ND-1000 spectrophotometer (Thermo Fisher Scientific Inc., Waltham, MA, US). The DNA concentration was measured by Qubit (Life Technologies, Stockholm, Sweden). DNA extraction and included RNAaseA treatment of DNA samples.

Bisulfite modification was performed using EZ DNA Methylation –Gold^TM^ Kit (Zymo Research, Cambridge CB23 8SQ, UK) on 500 ng of genomic DNA, according to the manufacturer´s instructions. Methylation levels at CpG sites were assessed in the bisulfite treated DNA samples by bisulfite pyrosequencing (PyroMark Q24 instrument, QUIAGEN). Percentage methylation was calculated by averaging across all CpG sites interrogated.

**Illumina 450K Data methylation analysis**

Methylation profiling of the samples was carried out using Illumina Infinium Human Methylation 450 BeadChip Kit (Illumina Inc.,USA), which interrogates 485,764 cytosine positions in the human genome; most of these, 365,934 CpG sites, are situated within known gene regions ‒promoter, gene body and UTRs (untranslated regions)‒ while 119,830 correspond to intergenic regions [1].

Briefly, one microgram of each sample was treated with sodium bisulfite using the EZ DNA Methylation Kit (Zymo Research, Orange, CA) following the manufacturer’s procedure, but with the modifications described in the Infinium Assay Methylation Protocol Guide, and this was followed by DNA hybridization to the BeadChip.

Raw IDAT files were obtained and pre-processed using the R/Bioconductor_minfi package [minfi] [2]. Red and green signals obtained from IDAT files and the SWAN algorithm were used to correct differences due to the different probe designs in the Infinium Human Methylation 450 array [3]. Probes that had been found to cohybridate with probes in the sex chromosomes [4] were also removed. We used the information from the SNP137Common track from the UCSC Genome Browser [5] in order to remove those probes with an SNP located inside their 2bp central region. Probes with detection *p*-values over 0.01 in at least two samples were filtered out. In accordance with Du et al [6], both β values and M-values were computed and employed across the analysis pipeline. M-values were used for all the statistical analyses, assuming homoscedasticity, while β values were mostly used for the intuitive interpretation and visualization of results.

In order to identify CpG sites which were differentially methylated (dmCpGs) between the different VAT samples, a robust moderated t-test implemented in the R/Bioconductor package limma [7] was used. False discovery rate (FDR) was controlled using the Benjamini-Hochberg procedure with a threshold value of 0.05. An additional threshold of effect size was applied, ensuring that only those probes with the greatest differences between groups were selected. The threshold was selected as the 30th percentile of the ordered absolute effect sizes, i.e., we kept the 70% of probes with the highest absolute difference between group means. The application of this threshold is essential to remove differences deriving from technical artifacts and consequently ensure a more biologically sound statistical data analysis [8]. Δβ was calculated for a given CpG site as the difference between the T2D and NT2D group means. A positive value indicates relative hypermethylation and a negative one, relative hypomethylation, S1 Fig.

**Genomic region analysis**

A probe was said to be in a promoter region when it was located inside the first exon, the 5′ -UTR or a region up to 2 kb upstream of the transcription start site (TSS) of any given transcript. Similarly, a probe found inside any intron or any exon other than the first was labeled as intragenic. Intergenic probes were determined as those which did not fall into either of the two previous categories. According to this definition, a probe could be in both a promoter and an intragenic region at the same time, for different transcripts. A contingency table was built for each selected subset of probes and a given genomic region, with one variable indicating whether or not a given probe belonged to the subset, and the other indicating whether a given probe was labeled as coming from the selected region. Significance of the association was determined by x^2^ -test. A significance level of 0.05 was used to determine if a subset was dependent with respect to a given genomic region. Odds ratio (OR) was used as a measure of effect size.

**CGI status analysis**

Shores were defined as 2 kb regions flanking CpG islands, and shelves as the 2 kb regions bordering the respective upstream and downstream shores. Probes not belonging to any of the regions thus far mentioned were assigned to the special category non-CGI. Each probe was assigned to only one category. A 4 × 2 contingency table was constructed for every subset of probes in order to study the association between the given subset and the different CGI categories. A Chi-squared test was used to determine if any of the categories had a significant association with the given subset. For each of the CGI status levels, a 2 × 2 contingency table was defined and another Chi-squared test was used to independently evaluate the association of the given subset with each status level, a significance level of 0.05 being employed for all tests. Effect size was reported as the odds ratio (OR) for each of the individual tests.

**Differential methylation analyses**

Statistical analyses were performed using R/Bioconductor (version 3.0; open source). To identify CpG sites showing differential methylation values between VAT samples a robust moderated t-test implemented in the R/Bioconductor package limma was performed [9]. p-values were adjusted by controlling the FDR (using the Benjamini-Hochberg method). Only those probes with adjusted p-values below a 0.05 significance level were selected.

**Microarray background correction**

Although it is sometimes referred to as a genome-wide solution, the HumanMethylation450 BeadChip only covers a fraction of the entire genome. In its 27K predecessor, the probes were mainly located at gene promoter regions, while in addition to the promoter probes; the HumanMethylation450 BeadChip includes probes located inside genes and in intergenic regions [10].

The irregular distribution of probes can lead to unwanted bias when studying whether a selected subset of probes is enriched with respect to any functional or clinical mark. A reference to the background distribution of features was included in every type of statistical test performed in order to prevent our conclusions from being driven by the irregular distribution of probes. In qualitative tests (CGI status, genomic region, or histone mark enrichment), the contingency matrix was built to represent the background distribution of the microarray. In quantitative tests (density of CpG, distance to centromeres and telomeres), the corresponding metric was compared between the subset of interest and the remaining probes in the microarray. Thus, any significant result would indicate a departure from the fixed background distribution and ignore any manufacturer bias.

**References:**

1. Sandoval J, Heyn H, Moran S, Serra-Musach J, Pujana MA, Bibikova M, et al. Validation of a DNA methylation microarray for 450,000 CpG sites in the human genome. Epigenetics [Internet]. 2011/05/20. 2011;6(6):692–702. Available from: http://www.ncbi.nlm.nih.gov/entrez/query.fcgi?cmd=Retrieve&db=PubMed&dopt=Citation&list_uids=21593595

2. Aryee MJ, Jaffe AE, Corrada-Bravo H, Ladd-Acosta C, Feinberg AP, Hansen KD, et al. Minfi: a flexible and comprehensive Bioconductor package for the analysis of Infinium DNA methylation microarrays. Bioinformatics [Internet]. 2014/01/31. 2014;30(10):1363–9. Available from: http://www.ncbi.nlm.nih.gov/entrez/query.fcgi?cmd=Retrieve&db=PubMed&dopt=Citation&list_uids=24478339

3. Maksimovic J, Gordon L, Oshlack A. SWAN: Subset-quantile within array normalization for illumina infinium HumanMethylation450 BeadChips. Genome Biol [Internet]. 2012/06/19. 2012;13(6):R44. Available from: http://www.ncbi.nlm.nih.gov/entrez/query.fcgi?cmd=Retrieve&db=PubMed&dopt=Citation&list_uids=22703947.

4. Chen Y, Lemire M, Choufani S, Butcher DT, Grafodatskaya D, Zanke BW, et al. Discovery of cross-reactive probes and polymorphic CpGs in the Illumina Infinium HumanMethylation450 microarray. Epigenetics. 2013;8(2):203-9. doi: 10.4161/epi.23470. Available from: https://www.ncbi.nlm.nih.gov/pubmed/23314698.

5. Sherry S, Ward M-H, Kholodov M, Baker J, Phan L, Smigielski E, et al. dbSNP: the NCBI database of genetic variation. Nucleic Acids Res. 2001;29(1): 308–311. Available from: https://www.ncbi.nlm.nih.gov/pmc/articles/PMC29783/

6. Du P, Zhang X, Huang CC, Jafari N, Kibbe WA, Hou L, et al. Comparison of Beta-value and M-value methods for quantifying methylation levels by microarray analysis. BMC Bioinformatics [Internet]. 2010/12/02. 2010;11:587. Available from: http://www.ncbi.nlm.nih.gov/entrez/query.fcgi?cmd=Retrieve&db=PubMed&dopt=Citation&list_uids=21118553

7. Smyth G, Gentleman R, Dudoit S, Irizarry R and Huber W. Limma: linear models for microarray data. Bioinformatics and Computational Biology Solutions using R and Bioconductor. New York: Springer; 2005. p. 397–420.

8. Pan KH, Lih CJ, Cohen SN. Effects of threshold choice on biological conclusions reached during analysis of gene expression by DNA microarrays. Proc Natl Acad Sci U S A [Internet]. 2005/06/14. 2005;102(25):8961–5. Available from: http://www.ncbi.nlm.nih.gov/entrez/query.fcgi?cmd=Retrieve&db=PubMed&dopt=Citation&list_uids=15951424

9. Ritchie ME, Phipson B, Wu D, Hu Y, Law CW, Shi W, et al. limma powers differential expression analyses for RNA-sequencing and microarray studies. Nucleic Acids Res [Internet]. 2015/01/22. 2015;43(7):e47. Available from: http://www.ncbi.nlm.nih.gov/entrez/query.fcgi?cmd=Retrieve&db=PubMed&dopt=Citation&list_uids=25605792

10. Dedeurwaerder S, Defrance M, Calonne E, Denis H, Sotiriou C, Fuks F. Evaluation of the Infinium Methylation 450K technology. Epigenomics [Internet]. 2011 Dec [cited 2017 Aug 10];3(6):771–84. Available from: http://www.ncbi.nlm.nih.gov/pubmed/22126295
